# RNA-seq data of the *Jatropha curcas* L. shoot system

**DOI:** 10.1016/j.dib.2018.09.081

**Published:** 2018-09-29

**Authors:** Nisha Govender, Siju Senan, Zeti-Azura Mohamed-Hussein, Mohd Noor Mat Isa, Zahira Yaakob, Wickneswari Ratnam

**Affiliations:** a School of Environmental and Natural Resource Sciences, Faculty of Science and Technology, Universiti Kebangsaan Malaysia, UKM, 43600 Bangi, Selangor, Malaysia; b Center for Bioinformatics Research, Institute of Systems Biology, Universiti Kebangsaan Malaysia, UKM, 43600 Bangi, Selangor, Malaysia; c School of Biosciences and Biotechnology, Faculty of Science and Technology, Universiti Kebangsaan Malaysia, UKM, 43600 Bangi, Selangor, Malaysia; d Malaysia Genome Institute, National Institutes of Biotechnology Malaysia, Ministry of Science, Technology and Innovation, Jalan Bangi, 43000 Kajang, Selangor, Malaysia; e Faculty of Engineering and Built Environment, Universiti Kebangsaan Malaysia, UKM, 43600 Bangi, Selangor, Malaysia

## Abstract

*Jatropha curcas* L. or the physic nut is a monoecious shrub belonging to the *Euphorbiaceae* family. The plant is an ideal feedstock for biodiesel production; oil-rich seed (37–42%), has a broad range of growth habitat such as arid, semi-arid and tropical and a relatively feasible process for conversion of crude oil into biodiesel. The major constraint affecting the success of large-scale *J. curcas* plantation is seed yield inconsistency. Numerous research projects conducted on *J. curcas* with integrated genetic, genomic and transcriptomic approaches have been applied on the leaf, apical meristem, flower, root and fruit tissues. However, to date, no genomics data of *J. curcas* shoot system are publicly available, despite its importance in understanding flowering, fruiting and seed set qualities targeted for yield improvement. Here, we present eighteen sets of shoot and inflorescence transcriptomes generated from *J. curcas* plants with contrasting yields. Raw reads of the RNA-seq data are found in NCBI׳s Sequence Read Archive (SRA) database with the accession number SRP090662 (https://www.ncbi.nlm.nih.gov/sra/?term=SRP090662). This transcriptomic data could be integrated with the present genomic resources for in depth understanding of *J. curcas* reproductive system.

**Specifications table**

**Table:** 

Subject area	*Biology*
More specific subject area	*Transcriptomics*
Type of data	*Table*
How data was acquired	*Ilumina HiSeq. 2500 platform*
Data format	*Raw: bam files*
Experimental factors	*Shoots and inflorescence*
Experimental features	*Parametric free*
Data source location	Experimental Plot A, Universiti Kebangsaan Malaysia (UKM), Bangi, Selangor, Malaysia (2°55′09.0′′N101°47′04.8′′E)
Data accessibility	*NCBI׳s Sequence Read Archive (SRA) database with the accession number*SRP090662
	https://www.ncbi.nlm.nih.gov/sra/?term=SRP090662

**Value of the data**

• The transcriptome data of shoot system reported here are closely associated to the reproductive component of the plant and therefore may provide an essential knowledge base for vegetative-reproductive transition in *J. curcas* at the molecular level.
• These *J. curcas* inflorescence transcriptomes may facilitate the molecular understanding of reproductive-related genes and gene functions in the shoot system.
• By using relevant bioinformatic approaches and functional studies, identification of candidate genes and critical component of pathways useful for genetic improvement of *J. curcas* shoot system could be done.

## 1 Data

The raw data (bam files) generated from the 18 sets of *Jatropha curcas* (shoots and inflorescences) transcriptomes has been deposited to NCBI׳s Sequence Read Archive (SRA) database with the accession number SRP090662 (https://www.ncbi.nlm.nih.gov/sra/?term=SRP090662). Description on the plants, total RNA extraction, sequencing and transcriptome construction is given in the next section.

## 2 Experimental design, materials and methods

### 2.1 Plant materials

Six individual plants (2-years-old) from four accessions (UKM JC-17, 18, 20 and 21), were selected from the Experimental Plot A, Universiti Kebangsaan Malaysia (UKM), Bangi (2°55′09.0′′N101°47′04.8′′E). Each plant was screened for number of fruits per plant. Two types of tissues were selected from the shoot system; i) shoot corresponding to the inflorescence (at 2.5 cm from the base of the peduncle) and apical meristem (at 2.5 cm from the tip of the apical meristem) and ii) inflorescence, a collective pool of male flowers, female flowers, pedicels and peduncle.

### 2.2 RNA isolation, library preparation and RNA-seq

Total RNA was isolated using the CTAB+silica column method [1], employing RNeasy Plant mini kit (Qiagen, Hilden, Germany) with minor modifications. The quality and integrity of total RNA were estimated using the NanoDrop ND*-*1000 Spectrophotometer (NanoDrop Technologies*,* USA) and a Bioanalyser RNA 6000 chips system (Agilent Technologies, USA), respectively. The cDNA sequencing libraries were prepared according to the SureSelect Strand-Specific RNA Library Prep for NGS Workflow protocol (Agilent Technologies, USA). The quality of the cDNA libraries was determined using the Bioanalyser DNA1000 (Agilent Technologies, USA). Genomic DNA fragmented by sonification was end-repaired and A-tailed using the polymerase activity of Klenow fragment. Sequencing was performed using the Ilumina HiSeq. 2500 (Yourgene Bioscience, Taiwan) with 100 bp-paired-end processing after validating the libraries by qPCR, Expersion and Qubit.

### 2.3 RNA-seq data workflow

The raw reads obtained from the 18 samples were subjected to pre-processing prior to further analysis. The raw fastq reads were trimmed using the Trim Galore (http://www.bioinformatics.babraham.ac.uk/projects/trim_galore/) package with the following settings; trim all paired-end reads with low quality ends with a Phred score less than 20, trim only the paired-end reads and introduce an Illumina adaptor sequence for any sequence that overlapped with at least 5 base pairs. The FastQc was run in the default settings on a FastQ file. All trimmed reads were aligned to *J. curcas* genomic sequences from Kazusa׳s Jatropha Genome Database version r4.5 (ftp://ftp.kazusa.or.jp/pub/jatropha/). The alignment was performed using the STAR aligner (https://github.com/alexdobin/STAR) and thereafter, was subjected to Cufflink tool (http://cole-trapnellab.github.io/cufflinks/papers/) to generate transcriptome assemblies. All packages were used with default settings. Descriptive statistics on the RNA-seq data of the 18 samples is given in Table 1.

**Table 1:** Statistics: RNA-seq data of the *Jatropha curcas* shoot system.

**Attributes**	**Values**	
	**Shoots**	**Inflorescences**
*Pre-processing*		
Total Ilumina raw reads	45,871,284–71,752,446	
*Post-processing*		
Sequence analysed (pair)	22,961,194–35,876,223	
Filtered (%)	3.49–4.75	
Aligner reads (pair) input	22,053,488–34,343,944	
Uniquely mapped reads (%)	81.02–88.22	
Reads mapped to multiple loci (%)	8.15–11.25	
*Transcriptome IDs*		
Biosample ID	SAMN05827464	SAMN05827465
	SAMN05827463	SAMN05827461
	SAMN05827462	SAMN05827457
	SAMN05827460	SAMN05827453
	SAMN05827459	SAMN05827451
	SAMN05827458	SAMN05827449
	SAMN05827456	
	SAMN05827455	
	SAMN05827454	
	SAMN05827452	
	SAMN05827450	
	SAMN05827448	
Reads mapped to gene model	16,886,160–26,833,093	
